# Electrochemical Devices in Cutaneous Wound Healing

**DOI:** 10.3390/bioengineering10060711

**Published:** 2023-06-11

**Authors:** J. Parker Evans, Chandan K. Sen

**Affiliations:** Indiana Center for Regenerative Medicine & Engineering, Indiana University Health Comprehensive Wound Center, Department of Surgery, Indiana University School of Medicine, Indianapolis, IN 46202, USA

**Keywords:** electrochemical, electroceutical, wound healing, diabetic foot ulcer, chronic wound, dressing, electric field

## Abstract

In healthy skin, vectorial ion transport gives rise to a transepithelial potential which directly impacts many physiological aspects of skin function. A wound is a physical defect that breaches the epithelial barrier and changes the electrochemical environment of skin. Electroceutical dressings are devices that manipulate the electrochemical environment, host as well as microbial, of a wound. In this review, electroceuticals are organized into three mechanistic classes: ionic, wireless, and battery powered. All three classes of electroceutical dressing show encouraging effects on infection management and wound healing with evidence of favorable impact on keratinocyte migration and disruption of wound biofilm infection. This foundation sets the stage for further mechanistic as well as interventional studies. Successful conduct of such studies will determine the best dosage, timing, and class of stimulus necessary to maximize therapeutic efficacy.

## 1. Introduction

Nearly two centuries ago, Emil du Bois-Reymond found ionic currents leaving skin wounds using a galvanometer [1]. This foundational work was not developed to therapeutic use until patterns of electric potentials within tissues and mechanistic links to genetic and biochemical pathways were identified [2,3]. Electroceuticals may be broadly defined as devices that manipulate electric currents or fields for therapeutic gain in healthcare. They are emerging as an interventional approach in wound care [4]. Here we summarize the theoretical bases of electroceuticals and current clinical applications of available technologies.

Electrochemical reactions are chemical reactions driven by, or resulting in, an electrical potential difference [5]. These reactions include the movement of ions through an ionically conducting and electrically insulating electrolyte and electrons through an electronically conducting phase between electrodes. Electrochemical signals are a dominant force directing cell migration in wound healing [6]. All cells regulate their resting membrane potential which regulates cell proliferation, differentiation, apoptosis, and migration [7]. Using novel and conventional electrophysiological techniques to precisely measure and manipulate these gradients in vivo has considerably expanded the understanding of large-scale, dynamic bioelectric characteristics [2,8,9].

Ion channel activity inside tissues causes slow, progressive changes in the tissue’s global bioelectric profile. In development, these bioelectric fields regulate the expression of genes that establish organs and limbs; they also create gradients of morphogens to align anterior–posterior and left–right axes [2,8,9,10]. Additional examples in *Drosophila*, Zebrafish, frog, *Planaria*, chick, and mouse demonstrate that perturbing the normal bioelectric field regulates organ and organism size [10,11,12,13,14].

Electrical signals play a crucial part in the operation of most organs, including the skin, and are commonly employed in the monitoring of the cardiovascular and central nervous systems. Recent reviews have discussed the functional roles ion channels play in development, cancer, regeneration, and wound healing [2,7,8,15]. Bioelectricity is particularly significant as a multicellular coordination mechanism. Bio-chemo-electric gradients are observable after the first cell division in vertebrate zygotes, and complicated bioelectric patterns are evident in *Drosophila* oocytes [2,9,10]. During the later stages of development, melanocytes and skin cells in general are sensitive to the bioelectric state of their neighbors [11,16].

**Figure 1:**
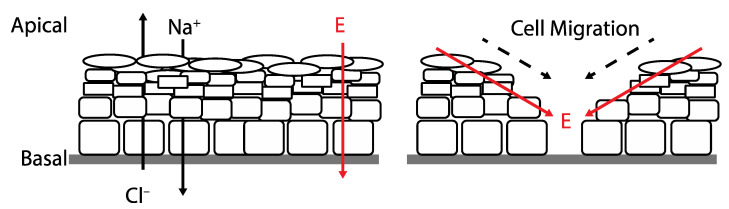
(**left**) Healthy skin with vectorial ion transport maintaining a transepithelial potential. (**right**) Disruption of the transepithelial potential caused by wounding and the subsequent effects on electric field and cell migration during wound healing.

A transepithelial potential (TEP) is found in nearly every major anatomical system. In smooth human skin, it is possible to detect TEPs of 30–100 mV and 0.5–10 mV across hairy skin [6,17]. These TEPS are created because sodium and chloride ion channels are disproportionately distributed at the apical end of the epithelial cells; in contrast, potassium channels are concentrated at the basal side while using an electrogenic transmembrane ATPase to increase intracellular potassium concentration at the same time that intracellular sodium concentration decreases. Lower sodium concentrations coupled with the negative TEP leads to Na^+^ influx into the cell and K^+^ efflux out of the cell. The resistance of intact skin is high due to the tight connections between cells that compose the monolayers of the epidermis and impede the flow of the current. After wounding, a current is driven through the wound’s low resistance route by the TEP (Figure 1). The resulting lateral electric field flows on both sides of the epidermis but with opposing polarity on each side (Figure 1). This suggests that the positive pole of the electric field is orientated toward the wound and the negative pole direct opposite [18]. The disturbance to the TEP is the initial signal created in response to a wound, and it is claimed to be responsible for commencing the process of wound healing by initiating keratinocyte migration, among other cell types, to the injured area via galvanotaxis [18].

Skin wounds breach the epithelial barrier, thus interrupting the TEP. As a result, the voltage near the wound becomes negative in comparison to the normal TEP [6]. Figure 1 shows that the endogenous potential gradient is laterally oriented, driving the current toward the wound. In skin wounds, the electric current flow is directed toward the lesion from the surrounding tissues and then away from the site, and the charge is offset by a cation flow in the opposite direction [6,19,20]. Spatial analysis reveals that the strongest physiological currents are in the wound’s periphery, whereas the wound’s center has a significantly weaker current flow [6]. Electric currents averaging 4 A/cm^2^ emerge temporarily at skin wounds soon after injury. Currents increase gradually to 10 A/cm^2^ within 120 min of injury and remain at 4–8 A/cm^2^ [21]. Similar magnitude currents are observed in corneal wounds but reach maximum flux at 60 min [6,22].

Away from the wound, cells continue to transfer ions to sustain the TEPs. Vectorial transport of ions is maintained by the apically positioned Na^+^/Cl^−^ pumps and the basally positioned K^+^ pumps in epithelia [6,20,21]. The electrogenic transportation system and potential differences are governed by anion secretion and cation absorption. Connected by tight connections, the outer cells of the epithelium constitute the principal electrically resistive barrier. The barrier and the directed ion transport establish the TEP [6]. These cells continue to generate electrical currents until the wound has healed and the standard TEP has been restored [6,23]. It is likely that the electrochemical effects in skin wounds and the subsequent responses are similar in other epithelia [6,17,19,24].

TEPs and wound electric fields are diminished by ion transport inhibition. Ouabain, benzamil, and furosemide may block the Na^+^–K^+^–ATPase, Na+ channels, and the Na^+^–K^+^–Cl^−^ transporter, respectively [25]. Alternatively, reduction of Cl^−^ in a bathing solution at the topical side increases the chemoelectric force pushing Cl^−^ to travel to the topical side, resulting in a substantial increase in Cl^-^ secretion [6,21]. This doubles the TEP and wound electric currents [6]. Conversely, decreasing [Na^+^] in the bathing solution reduces the chemoelectric forces that push Na^+^ to travel from the topical to the basal side of the skin, reducing TEPs and wound electric currents [6]. The application of medications with distinct modes of action that alter TEPs and increase or decrease the rate of wound healing suggest that in vivo cell migration and subsequent wound healing are governed by electrochemical reactions in the wound [6].

Chronic wounds, such as diabetic foot ulcers, tend to progress through the healing process at a slower rate than uncomplicated acute wounds, such as surgical incisions [26,27]. Chronic wounds most commonly stall in the inflammation phase of the wound healing process, prolonging or preventing advancement to the proliferative phase [23]. Additionally, more than 60% of chronic wounds are contaminated with bacteria that aggregate polymicrobially to form biofilms [28,29]. Pathogenic organisms infiltrate the wound tissue resulting in an increase in inflammation and harm to new tissue growth. Although some infected wounds may heal without intervention because of robust host defense systems, infected tissues should be treated as soon as possible to expedite the wound healing process and prevent complications, such as scarring, cellulitis, osteomyelitis, or septicemia [23].

Bacterial overgrowth of granulation tissue, characterized by a white or yellow coating, is occasionally observed in infected wounds [30,31]. Several variables and situations, such as impaired immune function, comorbidities, venous insufficiency, and inadequate circulation, may contribute to the development of a chronic wound through differing mechanisms [32]. The treatment of an infected wound differs from that of a non-infected wound in that the infection must be eradicated using oral or topical antibiotics; the wound must be drained or debrided to remove exudate or dead tissue, respectively, and antimicrobial dressings must be applied before the normal progression of wound healing phases can resume [23,30].

Many comorbidities contribute to the development of chronic wounds. A typical consequence of diabetes is peripheral neuropathy followed by foot ulceration [27,33]. In addition to peripheral neuropathy, peripheral artery disease, and peripheral ischemia are additional consequences which impact the proliferative phase of wound healing and result in a general slowdown in all wound healing phases [23]. Obesity is linked to an increased risk of ischemia and poor tissue oxygenation, which may result in slower wound healing or necrosis [34]. Unintentional tissue death is another factor that can impair wound healing; necrosis necessitates surgical debridement to remove the afflicted tissue before healing can continue. Malnutrition, notably insufficient protein consumption, is common in seniors and reduces blood vessel development, collagen production, and fibroblast proliferation, which eventually affects wound healing [35]. Nonsteroidal anti-inflammatory drugs (NSAIDs) reduce pain by inhibiting PGE2, a mediator of inflammation. NSAIDs are known to retard wound healing by inhibiting angiogenesis. Additionally, NSAIDs enhance scar formation, especially when administered during the proliferative phase [23]. The anti-inflammatory and immunosuppressive actions of steroids can inhibit wound healing by inhibiting fibroblast growth and collagen formation. Ionizing radiation can harm epithelial cells as they travel through tissues, resulting in skin tissue breakdown and impaired wound healing in patients undergoing radiation therapy [6]. Chemotherapeutic drugs influence the healing process by prolonging the inflammatory phase and reducing the synthesis of collagen. Cigarette smoking, specifically the usage of nicotine, restricts blood flow. Additionally, nicotine reduces the body’s immune response, which may increase the probability of wound infection [23]. Alcohol consumption is frequently coupled with poor dietary practices, which may combine to impair immunological function. In addition, alcohol may affect wound healing by reducing angiogenesis and collagen synthesis, resulting in the creation of weaker scar tissue and delayed healing overall [23,24,36].

The standard of wound care is to cover the injury with sterile dressings to prevent infection, inflammation, subsequent trauma, and to maintain moisture [30,36,37]. Historically, dressings consisted of cloth, cobwebs, dung, leaves, and honey [38]. Modern dressings consist of gauzes, polymers, gels, and foams [33,37,38]. Each material balances physical conditions at the wound site to optimize healing. At a minimum, dressings must be permeable to O_2_ and water vapor, maintain optimal temperature, debride dead tissue, and absorb exudate [33,36,38]. Some of these and other (physical, chemical, and biological) strategies could be applicable to treat biofilm infections. None have defined a new standard of care due to limited clinical evidence [39]. Burns and skin grafts are routinely treated with pressure dressings to prevent edema. To prevent additional complications, bandages can be designed to modulate a wound’s electrochemical environment, typically by incorporating electroactive materials into the dressings [40,41]. Electroceutical dressings are dressings with electroactive materials that modulate the electrochemical conditions of a wound [42]. The three types of electroceuticals reviewed here are ionic, wireless, and battery-powered dressings. Table 1 summarizes the devices reviewed by type and provides an illustration of the mechanism of action.

## 2. Methods

A literature search was performed in September 2022 to identify electroceutical wound care literature using the National Library of Health’s PubMed database. Search terms were determined by referring to top-ranked journal articles, guidelines from the Wound Healing Society, and review articles on wound management. Articles that were not available in English or which were not published in peer-reviewed journals were not considered. Studies were selected where descriptions of wound treatment in a clinical setting using portable devices that change the electrochemical environment directly at the wound and were used with, or were structurally part of, a dressing/bandage. The best investigations were adequately powered, with extended (>4 weeks) follow-up times, and used complete wound closure as a primary outcome. However, all abstracts meeting the inclusion criteria were screened, and full-length articles/reports were reviewed in depth, even if the study design could have been improved.

## 3. Results

### 3.1. Dressings Containing Metal Ions

Topical application of silver, copper, and zinc via metal-containing wound care dressings has positively impacted wound healing [56,69,70]. When compared to traditional bandages, those which sustain ion release require less frequent dressing changes, improving thermal insulation and patient quality of life, while decreasing the cost of care [42,71]. Metal ions are primarily used to prevent or treat wounds infected with bacteria, especially biofilm forming species. The presence of a large bolus continuously releasing ions increases the rate at which bacteria are eliminated [43]. There are metal ion-based dressings that do not rely on the release of the ions but rather work on electrical principles, i.e., generation of an electric field or current as anti-microbial and anti-biofilm treatment options [39,42,72]. Silver-based dressings are the most common metallic wound dressings found in the literature. Copper and zinc dressings were also included; however, no clinical results met the inclusion criteria. Dressings containing bismuth tribromophenate were not included because the mechanism is purely bacteriostatic [73].

### 3.2. Devices

Silver sulfadiazine is potentially the most commonly used silver formulation, particularly for burns [56,74]. The development of nano-crystalline silver dressings promotes continuous release of silver ions into the wound [44]. Four silver dressings appeared most often in clinical studies: Acticoat (Smith + Nephew) is a nano-crystalline silver dressing that continuously releases large amounts of silver. Actisorb^TM^ silver (3M) and Aquacel^®^-Ag (Convatec) absorb wound exudate while releasing a small amount of silver from the dressing. Contreet Foam (Coloplast) does both by optimizing the surface silver concentration while sequestering exudate from the wound surface into the bandage. This has the added benefit of increasing the bacterial exposure time to silver within the dressing. Urgotul (Urgo Medical) is a less potent form of silver sulfadiazine [43,44,53,56,56,57].

MedCu bandages are soft, single-use wound dressings where copper oxide microparticles infiltrate all layers [62]. Dressings are supplied in a sterilized pouch, with or without an adhesive backing, and are fabricated from an absorbent layer and 1–2 layers of orange polypropylene which contacts the wound bed. The absorbent layer is needle-punched, and the polypropylene is nonwoven. MedCu may be folded or cut to match the wound size and secured with Tegaderm^TM^ (3M). Bandages are viable for seven days or until saturated [63].

Metcovazin impregnated gauze is commonly used as a primary wound contact dressing in Indonesia [65]. Metcovazin is an inexpensive, topical, zinc-based cream that promotes moisture balance within the wound and allows easy removal of the old dressing [75]. It is locally produced from chitosan, petroleum jelly, and zinc. Zinc cream has several unique features as it not only promotes a moist wound environment but also facilitates tissue regeneration [24,66,67]. The Unna Boot Zinc Oxide Compression Dressing (Dynarex) is a similar device wherein the Metcovazin is impregnated into a compression garment [68].

### 3.3. Mechanism

**Figure 2:**
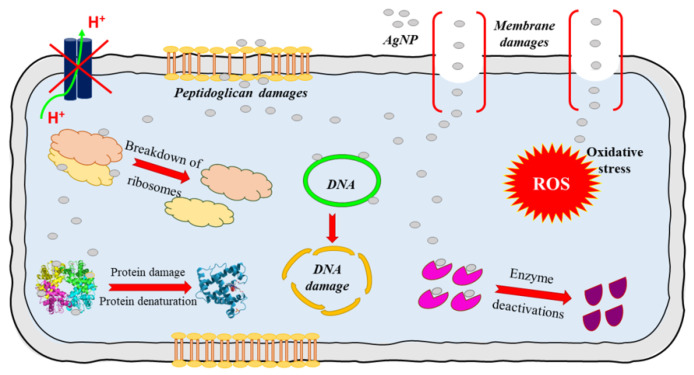
Mechanism of action of silver nanoparticles (AgNP). Reproduced with permission from Rybka, M., Mazurek, Ł., Konop, M., Beneficial Effect of Wound Dressings Containing Silver and Silver Nanoparticles in Wound Healing—From Experimental Studies to Clinical Practice. Published by MDPI, 2022 [76].

Silver’s benefit as a wound therapy has been recorded since 69 B.C [44]. Recently, silver has attracted increased study as a topical antibacterial agent. Silver metal (Ag) has no therapeutic properties, but the silver ion (Ag+) is cytotoxic to bacteria, viruses, yeast, and fungi [74]. When an ion-releasing bandage is applied to a wound, an electrochemical gradient drives silver ions into the wound and any bacterial cell walls. The electrochemical gradient is comprised of a concentration gradient, which will always favor the ingress of Ag+ into the cell because of the low intracellular silver concentration and an electric potential gradient which can change with local silver concentration. Figure 2 shows that in areas of relatively low electrochemical gradient, some ions may compromise a bacterium’s cell wall and cell membrane function by irreversibly binding to ion channels and disrupting the peptidoglycan layer [76]. Where the local electrochemical gradient is higher, ions are driven into the cell where they irreversibly bind to the proteins in the electron transport chain. Additional damage may occur from permanent DNA binding which prevents transcription and halts the cell cycle.

Silver ions are electrostatically attracted to sulfur-containing macromolecules. Thus, silver ions are toxic to numerous metabolic components of bacterial cells. Ag+ binds to DNA, RNA, and several proteins, resulting in cell death via multiple mechanisms, including protein and nucleic acid denaturation, enhanced membrane permeability, and poisoning of the respiratory chain [47,74]. Compromising a bacterium’s cell wall and/or membrane permeability results in cessation of ion transport, alteration of proteins, halting of transcription, and disruptions to the cell cycle. Ionic silver inhibits microbes in the planktonic state; therefore, it is anticipated that Ag+ treatment during early biofilm development may be effective. However, the few in vitro studies testing Ag+ on mature biofilms identified that higher concentrations of silver are needed to disrupt mature biofilms [44,77]. The concern would be the toxic effects of such high concentrations on host cells when used in vivo [52], thus impairing wound healing. Preclinical porcine studies on a burn biofilm model showed that Ag+ has a limited effect on established biofilms, and the emergence of Ag+ resistant pathogenic strains is a potential concern [44].

Copper oxide impregnated wound dressings are approved for treating acute wounds but are efficacious on chronic wounds, namely diabetic, pressure, and venous ulcers. The REDOX couple between Cu_2_O and CuO delivers copper to the wound and creates hypoxia in the wound environment, which upregulates Hif-1 [62,63]. The overexpression of Hif-1 in the dermis and macrophages leads to endothelial cell migration and proliferation, formation of new blood vessels, fibroblast movement, additional extracellular matrix protein secretion, and enhanced epithelialization [23]. Copper oxide demonstrates potent, wide spectrum antimicrobial effectiveness (>4 log reductions) for 7 days, even when dressings are saturated with wound exudate; antimicrobial effectiveness against *E.coli*, *E. faecalis*, *E. aerogenes*, *K. pneumoniae*, *S. epidermis*, methicillin-resistant *Staphylococcus aureus*, and *C. albicans* is maintained after 7 days of consecutive microorganism inoculations [62,63]. In the context of antibiofilm activity, copper oxide nanoparticles have primarily been tested in vitro and shown to have dose-dependent, differential effects against Gram positive vs. Gram negative strains of biofilm forming bacteria [78,79,80].

In contrast to silver and copper, the largest concentration of zinc in the skin is found in the keratinocytes closest to the basement membrane responsible for maintaining the TEP [6,69]. During the early phase of inflammation, zinc levels rise along the wound’s margins, and this concentration rises during the granulation and epithelialization phases. This is the result of enhanced membrane transporter expression in keratinocytes, fibroblasts, and macrophages. The last phase of wound healing is marked by a reduction in cell migration [69]. Zinc applied topically to wounds enhances autolytic debridement due to the enhanced collagenase activity of matrix metalloproteinases. This advantage has been reported in case studies of patients with diabetic foot, pressure ulcers, and burns. Zinc aids in the protection against apoptosis by acting as the cofactor for antioxidant proteins that prevent damage from reactive oxygen species and bacterial toxins [67]. In vitro studies demonstrated that various bacterial species are inhibited by zinc. This also explains the ability of zinc to prevent biofilm formation [81]. However, methicillin resistance, among other strains, is evident and zinc’s capacity to inhibit biofilms is limited [24,69]. A recent study in a mouse full-thickness excisional wound model infected with a biofilm-forming *S. aureus* strain also appears to corroborate the ability of zinc to prevent biofilm infection and therefore promote wound healing either alone or in synergy with Gentamicin [82]. Treatments were performed at the time of infection; therefore, it is likely that the treatments targeted planktonic bacteria. It remains unclear if such treatments could disrupt preformed biofilms.

### 3.4. Clinical

Most experimental and clinical support for silver dressings has limited patient populations and is non-comparative. The absence of primary evidence is partially compensated for with in vitro, experimental, and small cohort clinical studies. While informative, these studies are limited because some were financed by industry, and few were comparative. For most products, the selection of a silver dressing rests mostly on this limited evidence [43,44,47,48]. Numerous case reports present the use of copper and zinc dressings, but they did not meet the inclusion criteria.

Khansa et al. systematically reviewed 59 papers where silver was used for wound treatment [44]. The authors discovered that silver nano-crystalline bandages were beneficial for contaminated wounds, and that pain was reduced because fewer dressing changes were required when compared with the standard of care (SOC). For the treatment of burns, silver-containing bandages demonstrated better epithelialization, pain levels, and overall treatment cost over SOC. Silver dressings were found to be advantageous when initially treating infected wounds. However, no advantage was found in non-contaminated wounds.

In a review of 70 patients with burns treated using nano-crystalline silver dressings, fewer occurrences of cellulitis and antibiotic use were noted [49]. Studies lacking an adequate control group have demonstrated safe application practices and reduced burn pain. Comparing healing of meshed skin grafts treated with nano-crystalline silver dressings vs. topical antibiotic creams revealed that the 20 patients treated with dressings saw superior graft epithelialization. Further, nano-crystalline bandages were superior to silver sulfadiazine in preventing burn wound infections by bacteria. In a randomized clinical experiment including 30 patients with burns, the antibacterial efficacy of nano-crystalline silver technology was compared to that of silver nitrate. The dressings were deemed simple to apply and less painful, with less infection as evaluated by biopsies and a lower frequency of bacteremia [83].

In phase II noncomparative research involving 24 patients with mid-thickness burns, 22 of whom were evaluable, Aquacel^®^-Ag performed well in terms of patient compliance [84]. In a 4-week study including 30 patients with chronic, mixed venous, diabetic, or pressure ulcers which had failed to heal from SOC, the administration of Aquacel^®^-Ag decreased wound area and discharge, had higher patient comfort levels, and improved granulation tissue quality [54]. Other studies indicated that the wound area was reduced after Aquacel^®^-Ag treatment, but bacterial counts remained static [55]. In larger, non-powered comparison research involving 99 patients from 13 centers, Silvercel was compared to Algosteril in ulcered patients [53]. In the brief 14-day observation period, patients undergoing silver dressing treatments were linked with fewer clinical infections requiring antibiotic therapy.

In a noncomparative study including 25 patients with leg ulcers, Contreet Foam safely and effectively managed wound fluid for one month. In a comparative 6-week study with Biatain, Contreet Foam was more effective for the treatment of diabetic foot ulcers. In critically colonized chronic venous ulcers, Contreet Foam outperformed comparable non-silver foams, according to a randomized, non-powered, multicenter study. During the four-week trial period, the size of ulcers treated with Contreet Foam decreased dramatically, with fewer bandage changes, leaks and less odor.

Silver sulfadiazine cream has beneficial effects on chronic wounds by inhibiting microbial development and treating infection. However, there is no consensus regarding silver sulfadiazine’s role in managing infections in chronic wounds. Khansa mentioned findings demonstrating silver sulfadiazine’s negative effect on burn patient infections, epithelialization, and scarring. Silver sulfadiazine initially emits up to 3176 ppm of silver into the wound, which is harmful to mammalian cells. However, the concentration of silver in the wound rapidly drops below harmful levels and subsequently, to the therapeutically effective levels. Khansa et al. reached the conclusion that silver sulfadiazine should not be utilized for wound or burn treatment [52].

No trials using MedCu or other copper dressings met the inclusion criteria, but NCT04634838 is currently recruiting [85]. Few randomized controlled experimental trials have been undertaken to evaluate zinc’s efficacy in wounds. Zinc-deficient patients must take zinc sulphate supplements orally to promote proper wound healing. However, if normal levels exist, a systematic clinical trial evaluation involving lower extremity wounds concluded that there is no effect above placebo [69]. However topical zinc treatment has showed experimental and clinical improvement in patients with deficient and normal serum levels. A randomized clinical trial involving 113 patients with venous ulcers contrasts three treatments for leg wounds: zinc paste bandage, zinc stockings, and a calcium alginate dressing [69]. In every instance, the zinc bandages were secured with a compression bandage. The faster healing observed in the zinc-treated patients is attributable to the effects from compression, but the possible benefits of topically applied zinc cannot be ruled out.

### 3.5. Limitations

Like other antiseptics, ions are made inert when bound to proteins. This can have therapeutic effect, but it can also be caused by tissues, anions, and beneficial proteins. Complications from ions in wound therapy are minor and primarily focused on silver [44]. Local staining by silver dressings is common but is a minor complication and is indicative of sustained release [44,53,56,74]. Although the shade of pigment is related to the [Ag+] at the wound’s periphery, tissue penetration is minimal [48]. Systemic toxicity, argyria, is uncommon with ionic silver and silver metal [44]. Nevertheless, it is possible that argyria could result when dressings that sustain the release of concentrated doses of silver are used on burns or wounds with high surface area. Silver allergy in humans is reported inconsistently [44,48,56].

*Staphylococcus* and *Pseudomonas* are prevalent in chronic wounds and have notable antibiotic resistance; however, silver has retained its high antibiotic activity even against these common pathogens [43,47]. Resistance is less likely to develop against silver than traditional antibiotics because the silver targets multiple bacterial systems. Despite the lack of resistance to silver, it is still possible for a microbe to develop resistance upon multiple exposures to silver.

Several published studies claim antimicrobial effects of metal ions [39,77,78,79,80,81,82]. Very few of these claims hold up against biofilm forming microbial strains. A significant gap in understanding relates to much of these studies being performed in vitro and/or during the early stages of biofilm development, when microbes may still be in the planktonic mode of growth. There is a strong need for relevant in vivo and clinical studies that specifically test metal ions against preformed biofilms (particularly of mixed microbial species).

### 3.6. Wireless Electroceutical Dressings

Wireless electroceutical devices are soft, single-use wound dressings with small, dispersed deposits of at least two different metals. The metals form a REDOX couple when ionically connected by wound fluid or another electrolyte [57]. Dressings can be used until the REDOX couple reaches equilibrium. Interestingly, Ag+ and Zn^2+^, which are ions with therapeutic effect, are used to create REDOX couples in wireless electroceutical devices.

### 3.7. Devices

Procellera^TM^ is a woven fabric bandage with screen printed microelectrodes intended to treat partial or complete thickness wounds [46,57]. It generates 2–10 mV between individual microdeposits of Ag and Zn metals contained within a woven fabric which form a REDOX couple upon exposure to moisture. It yields 0.6–0.7 V across the wound at a current of ~10 microamps [57]. Another wireless silver/zinc device was reported by Yu et al. with a similar structure [58].

**Figure 3:**
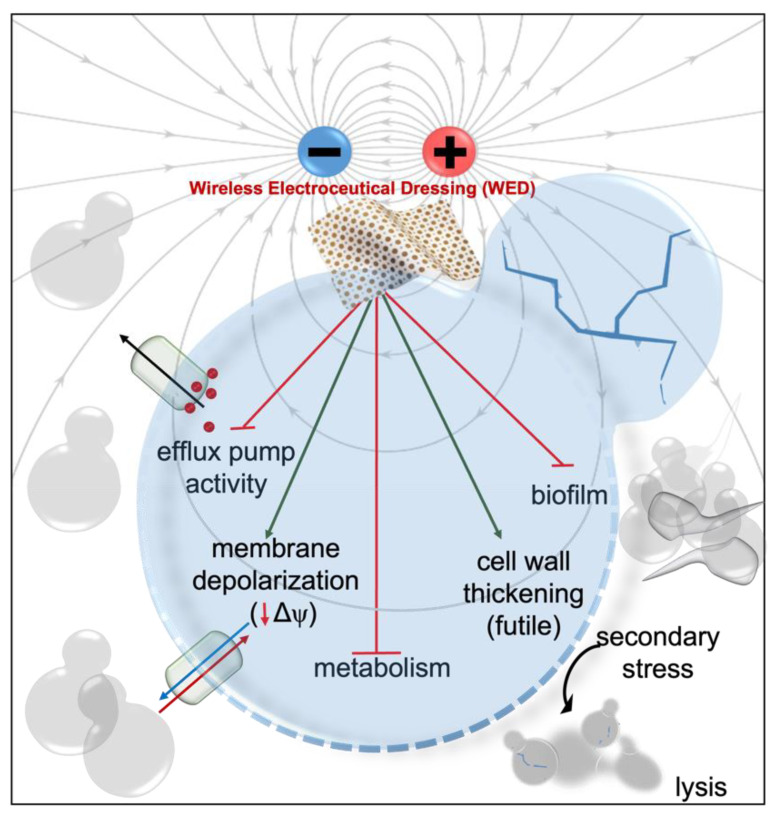
Mechanism of Procellara. Reproduced with permission from doi:10.1016/j.bioelechem.2021.107921. This article was published in Bioelectrochemistry, 142, Khona, D.K., Roy, S., Ghatak, S., Huang, K., Jagdale, G., Baker, L.A., and Sen, C.K., Ketoconazole resistant Candida albicans is sensitive to a wireless electroceutical wound care dressing, 107921, Copyright Elsevier (2021) [86].

Figure 3 shows the complex mechanism of wireless electroceuticals due to the interactions between electrochemical fields from the wound and the REDOX couples [86]. In one in vitro investigation, exposure to a silver/zinc bioelectric dressing enhanced keratinocyte migration, but treatment with Ag or Zn alone had no effect [45]. The researchers discovered that this bioelectric dressing formed modest amounts of non-toxic H_2_O_2_, which preserved a pathogen-free wound environment [45,57].

Studies demonstrating the attenuation of electron paramagnetic resonance spectra indicate that wireless electroceuticals spontaneously generate superoxide when connected by a salt bridge [45,57]. Superoxide generation is responsible for neutrophils’ “respiratory burst” at the wound used to destroy pathogens [23]. The dressing’s capacity to continuously maintain low superoxide concentrations at the wound aids in maintaining a wound environment free of pathogens. The superoxide anion is a strong reductant which can suppress the transcription of critical bacterial operons essential for quorum sensing, the Mex efflux pump, glycerol-3-phosphate dehydrogenase activity, and pyocyanin production [37,42,45,57,73].

### 3.8. Clinical

Electroceuticals were commonly used after extended SOC had failed [87]. In rare research on clean wounds, Blount et al. administered the Procellera^TM^ bandage to 50% of the donor region for all 13 patients undergoing skin grafting. They observed enhanced wound healing, scarring, and subjective patient outcomes.

A controlled, randomized, clinical trial tested different treatments for blisters resulting from Special Operations training [88]. Eighty United States Army Ranger trainees suffering from blisters on the lower extremities were randomly assigned (*n* = 40 per group) to receive Procellera^TM^ or the SOC. The study evaluated the healing rate of Procellera^TM^-treated blisters vs. the SOC. The study also evaluated wound exudate for biomarkers and infectious microorganisms. Nearly ¼ (9 out of 38) patents had bacteria in the wound exudate collected prior to treatment. Comparative samples were not collected in either arm because the wounds had closed prior to the second collection point. However, Procellera^TM^ dressings can be changed less frequently, which may impart tactical or strategic advantages over the current SOC in military medicine.

Whitcomb et al. evaluated Procellera^TM^ against the SOC for patients suffering from dehiscence and various lower extremity ulcers [89]. On average, patients were older than 80 and suffered from pre-existing conditions. In this fragile patient population, the Procellera^TM^ arm healed nearly twice as fast (19.8 d vs. 36.3 d, *P* < 0.05) and wound area decreased faster and more consistently. The wound area of the SOC arm increased before improvement was noted. Das Ghatak et al. met with positive outcomes in high-risk demographics treated with Procellera^TM^, especially patients with diabetes [42].

### 3.9. Limitations

Wireless electroceuticals are broadly applicable to various ulcers (diabetic, venous, pressure) and wound thicknesses. Their attractive, practical features, including patient safety, low cost, and long shelf life, urge additional in vivo research. Larger studies are needed to assess the ability of wireless electroceuticals to promote wound healing when compared to the SOC. Wireless electroceuticals with different/additional REDOX couples should be explored.

### 3.10. Battery Powered Dressings

**Figure 4:**
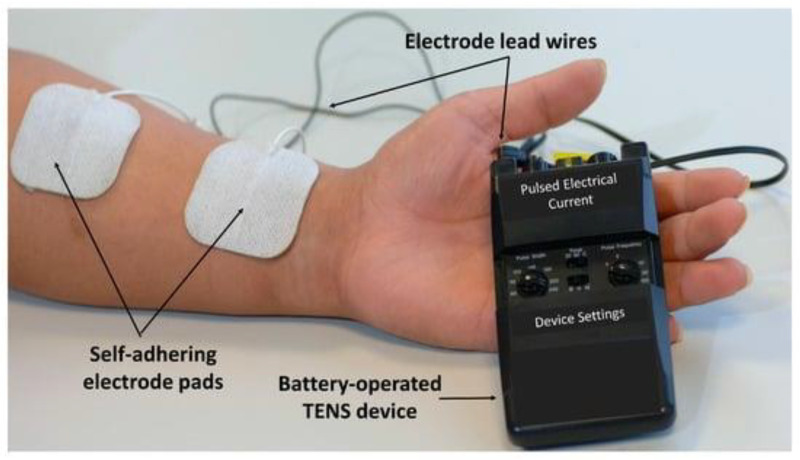
TENS equipment. Reproduced with permission from Johnson, M.I., Resolving Long-Standing Uncertainty about the Clinical Efficacy of Transcutaneous Electrical Nerve Stimulation (TENS) to Relieve Pain: A Comprehensive Review of Factors Influencing Outcome; published by MDPI, 2021 [90].

Devices which use a battery to impose an external electric field are the final type of electroceutical covered in this review. These devices vary considerably in design, but all operate with a constant input voltage source enabling varying levels of programmability [19]. Often, transepidermal nerve stimulation (TENS) devices (Figure 4) are included in this class of device, but this review will focus on devices purpose-built to promote cutaneous wound healing [90].

### 3.11. Devices

The patterned electroceutical dressing (PED) is fabricated from skin-safe silk, Ag/AgCl ink, and a waterproof support [42]. Power is provided by a 6 V battery in a waterproof polymer and current is limited by a 10 kΩ resistor. The maximum power density measured was 60 μW/cm^2^ at the anode and 753 μW/cm^2^ at the cathode, more than satisfying the FDA power density limit of 0.25 W/cm^2^ [42].

PosiFect RD^®^ is a similar battery-operated bandage [6,19,50,51]. PosiFect RD^®^’s electrode configuration is uniquely comprised of a round electrode applied to the wound, as in the other electroceutical reviewed, and another electrode placed concentrically around the wound’s periphery.

Accel-Heal operates by placing two electrode pads attached to the Accel-Heal power unit to the skin near the wound. The device sends a pre-set, automated regimen of subsensory electrical pulses to the wound to stimulate normal healing with the simple press of a button [59]. Once the dressing has been placed and the device has been activated, electrical stimulation is automatically provided to the wound for 48 h without additional intervention. Throughout the 12-day therapy period, the Accel-Heal electrodes must be replaced every 48 h.

WoundEL^®^ is a battery-powered electrostimulation system meant to expedite wound healing and alleviate wound-related discomfort [19]. Dressing electrodes are 190 × 130 × 45 mm and weigh 600 g. By default, the console is configured to oscillate between negative potentials at 128 Hz. The purpose of negative polarity is to encourage debridement and granulation. To promote epithelialization, the device can be switched to oscillate between positive potentials at 128 Hz. These settings are suggestions and physicians may prescribe and enact different treatments with the same device.

### 3.12. Mechanism

Battery-powered dressings have variable voltages, currents, modes, electrode layouts, and application durations [19,50]. Several distinct electrical waveforms and modalities are used, such as direct current (DC), alternating current (AC), and pulsed current (PC); each waveform creates distinct chemoelectric gradients within the wound [5,19]. Due to the cellular membrane’s strong resistance to ionic currents, ions surround the cells and create an electrochemical gradient across the cell. This potential gradient stimulates intracellular signaling pathways by inducing chemoelectric changes in the membrane proteins’ structure/function and activating ion channels [17,40]. The signaling cascade modifies the expression of the genes encoding proteins with multiple biological activities, including cell division, migration, and proliferation [23,53].

### 3.13. Clinical

To determine if PED was safe and efficacious for human use, a small trial was carried out to identify the effects of PED application on hosts who would be undergoing amputation [42]. Patients with one or more wounds on the affected leg were enrolled in this pilot trial. The study’s primary objective was not to save the lower extremity but to determine if PED treatment was safe for human use. One of eight patients was treated with the PED and a 1 kΩ resistor. Two days after the initial treatment, at the first dressing change, skin irritation was observed. All other participants were administered the PED with a 10 kΩ resistor, and no additional adverse events were reported. One other case of skin discoloration was attributed to silver staining. PED-10 was safe and effective on the eight amputees, and the observations described here suggest it should be explored in a larger cohort of chronic wounds. A Department of Defense-funded clinical study (NCT04794621) is currently testing these dressings in this very context and is expected to conclude in early 2023.

Several clinical studies that employed the woundEL^®^ pulsed current device for chronic wound treatment revealed positive outcomes [52,91,92]. A high-voltage pulsed current utilizes a double-pulsed monophasic pulsed current. Each pulse has a length of less than 200 microseconds and peak voltage between 150 V and 500 V. A high-voltage pulsed current has been utilized for wound healing, pain alleviation, and edema reduction.

Peters et al. conducted a 12-week randomized controlled experiment on 40 diabetic individuals with chronic ulcers on their feet [93]. Random assignments were made for patients to receive either a high-voltage pulsed current or a placebo. Throughout the 12-week trial, treatment was carried out every day with hourly sessions of 20 min for 8 h per day. Healing rates in the electroceutical group were 6% vs. 35% for those treated with the placebo. These results were not statistically significant (*P* = 0.058). A result of the study that did reach significance was that patients who frequently used (3X or more per week) the device were significantly more likely to heal than those who used the device fewer (0–2) times per week (*P* = 0.038).

A randomized controlled trial recruited 27 patients with 42 (arterial, venous, chronic) ulcers on their lower extremities. Assignments to treatment or placebo groups were carried out randomly [93]. The 150 V, 100 pps treatments lasted 45 min three times each week for four weeks. The treatment group’s wounds were much smaller (44%) than those of the placebo arm (16%). However, after 4 weeks, the benefits were no longer significant. Another randomized controlled trial compared an ischemic wound treatment using this technique to sham therapy over a 3.5-month period and found that wound area decreased and circulation improved. A final high-voltage pulsed current trial involved sixty participants with chronic pressure ulcers divided into four groups. One received a placebo, while the other three received a high-voltage pulsed current for 45, 60, and 120 min daily for one week. Wounds were measured at 0 weeks, 3 weeks, and 5 weeks, and a substantial reduction was observed in the groups that underwent high-voltage pulsed current for 60 or 120 min. There were no significant changes between the therapy groups, however.

The review by Hampton and coauthors highlighted two cast studies of chronic leg wounds that did not heal with the SOC; subsequent treatment with PosiFect RD^®^ achieved total wound closure in less than 3 months [61]. In a comparative clinical trial, wounds treated with Accel-Heal fully healed in ~10 weeks compared to 14 weeks in the SOC group. Notably, the wounds in the Accel-Heal arm had wounds that were 1.4 years longer than the SOC arm. An intermittent low-intensity direct (LIDC) current pulses an electric current between 0 and ~30 mA [60]. A double-blind multicenter controlled trial compared LIDC’s effectiveness in 43 patients with advanced pressure ulcers to 31 patients undergoing SOC. The current was varied between 300 and 600 mA. Twenty-seven patients did not respond to the SOC while four patients in the SOC group experienced a maximum of 80% wound closure. In the LIDC arm, complete wound closure was observed in 25/43 (58%). The authors reported that LIDC was significantly (*P* = 0.001) more effective in inducing total wound closure than the SOC. These favorable results imply intermittent LIDC treatment may be beneficial in chronic wounds. The trial does suffer from a failure to randomize subjects and does not explain the size disparity between the treatment and SOC groups. Feedar and colleagues used LIDC in a double-blind multi-centered randomized controlled trial involving fifty ulcers on 47 patients randomly assigned to SOC or treatment arms [6]. LIDC was administered at a rate of 35 milliamps for a duration of 30 min, twice daily. The treatment group’s healing rate of 56% was significantly (*P* = 0.04) faster than the 33% observed in the SOC arm [64].

These encouraging results suggest that additional, well-designed clinical trials are justified to optimize battery-powered electroceuticals to reduce the infection load associated with cutaneous wounds in clinical settings. These trials will have to overcome the currently held belief that electroceuticals can only be used to control infection in chronic wounds.

### 3.14. Limitations

An inherent consideration for all battery-powered devices is battery lifetime. Several authors have hypothesized that patient compliance is a primary factor that influences wound healing with battery-powered devices [6,19]. Most trials were administered in a clinical setting; consequently, attendance at clinic appointments was the primary compliance indicator. Malin et al. highlighted the unique compliance challenges electroceutical patients may encounter when compared to the SOC, namely daily battery recharge reminders [94]. However, compliance rates between treatment and SOC arms were similar, and neither group suffered any adverse effects. In both arms, some patients were removed for failure to adhere to the protocol. It quickly becomes apparent that electroceuticals with energy storage solutions have distinct advantages for patient compliance. Peters et al. evaluated a home-use device that measured patient compliance by logging the duration of use [93]. Compliance rates were comparable between the treatment and sham groups. Compliant patients in the electroceutical treatment group had a greater rate of wound healing (71%) when compared to non-complying patients in the electroceutical arm (50%) and all patients in the sham arm.

## 4. Conclusions

Bioelectrochemical fields are foundational signals that guide cell movement and influence wound healing. Electroceuticals can be divided into ionic, wireless, and battery-powered devices. Evaluations of electroceuticals mostly occur in chronically wounded patients, such as those with pressure ulcers, venous ulcers, vascular ulcers, and diabetic foot ulcers. All three classes of electroceutical devices can externally modulate electrochemical signals affecting molecular charge distributions to modulate biological function. Most clinical studies demonstrated improved cell migration, wound healing rates, and enhanced local perfusion when compared to SOC or sham therapy. The variation in outcome assessments, the kind of electroceutical, and dosage in the clinical studies is one of the limitations of this review. Additionally, most studies were small, and several used brief follow-up time points. In addition, the primary endpoint of several of the studies was a change in the wound’s surface area instead of total wound closure, primarily due to limited timeframes.

Current instrumentation challenges include the development of electrode systems that are disposable, economically viable, clinically resilient, and promote patient compliance. In well-designed systems, these obstacles are surmounted, and the cost of the equipment is compensated for by speeding the healing process and avoiding comorbidities. Clinical study design is critical to advance electroceuticals, as instrumentation requirements are unlikely to impede the advancement or adoption of electroceutical technology. Currently, a randomized open-label clinical trial testing a sequential electroceutical strategy in infected or high-risk burn, trauma, surgery, or chronic wounds is recruiting (NCT04794621). The design of this trial is powerful in that its primary endpoint is total wound closure, and it combines two different classes of electroceuticals.

Future directions include the use of electroceuticals in dental and orthodontic complications. Electroceuticals enhance cellular activity, facilitate tissue regeneration, and accelerate the healing process in the oral mucosa of patients [95,96,97]. This will likely benefit patients after hygiene procedures aid in preventing mucositis and improve bone orthodontic movement. Following oral hygiene procedures, electroceuticals can aid in reducing inflammation, minimizing discomfort, and promoting faster recovery [97]. In the context of preventing mucositis, which is inflammation of the oral mucosa often associated with chemotherapy or radiation therapy, electroceuticals can help mitigate the severity of mucositis, decrease pain, and enhance the healing of oral tissues [96]. Additionally, electroceuticals have shown promise in orthodontics as they may facilitate improved bone remodeling and rate of space closure in the distraction area [95]. Overall, electroceuticals stand to provide a valuable therapeutic approach in oral care, promoting healing, preventing complications, and improving treatment outcomes.

Overall, the evidence to date suggests that electroceuticals are an excellent adjunct therapy to the standard of care, but additional clinical trials will optimize dosage, timing, and type of stimulus to be administered to maximize therapeutic effectiveness. Few device-attributable problems or adverse effects have been recorded, showing that the treatment is both safe and simple. An interesting direction for further research is improving bone healing in oral implantology [98]. Specifically, this may aid with tilted implants in diabetic patients by diminishing microbial contamination around the oral cavity [98,99]. Lingering questions include the optimal method of delivering electrochemical stimulation to each wound type, the optimal electrode configuration, frequency, and duration for each combination of electroceutical and wound type. These questions must be addressed with large clinical trials evaluating various electroceuticals, treatment durations, and application frequencies on total wound closure.

## Figures and Tables

**Table 1:** A comparison table of the devices under review by category. The black arrows indicate the direction of the electric field for each electroceutical. Dressings are universally represented by a cream-colored substrate; silver, copper, and zinc are represented as their elemental colors; wound fluid is depicted as blue; and harmful bacteria are green.

Ionic		Wireless		Battery-Powered	
The electrochemical gradient drives ions to the bacterial cell. Where the local electrochemical gradient is high enough, ions are driven into the cell where they disrupt the respiratory system and bind to DNA.		The electrochemical potential difference creates an electric field that is maintained by ion transport between the anode and cathode until the electrochemical reaction reaches equilibrium.		An electric potential is applied to an inert electrode and the field is maintained until the battery depletes.	
**Commercial name**	**Reference(s)**	**Commercial name**	**Reference(s)**	**Commercial name**	**Reference(s)**
Silver sulfadiazine	[43,44]	Procellera	[45,46]	Patterned electroceutical dressing	[42]
Acticoat	[47,48]				
Actisorb	[49]			PosiFectRD	[6,19,50,51,52]
Aqucel-Ag	[53,54,55]				
Contreet Foam	[43,44,53,56,57]	Yu et al.	[58]	Accel-Heal	[59,60,61]
Urgotul	[43,44,53,56,57]				
MedCu	[62,63]			WoundEL	[60,64]
Metcovazin	[24,65,66,67,68]				
**Mode of action**		**Mode of action**		**Mode of action**	
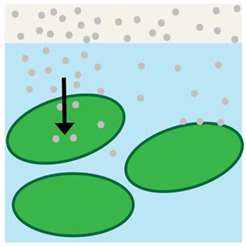 Silver circles represent silver ions, green ovals represent bacteria, and black arrows represent the electrochemical gradient.		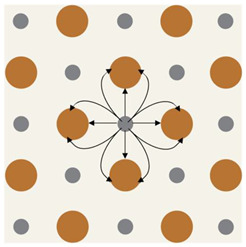 Silver circles represent silver electrodes, copper circles represent copper electrodes, and black arrows represent the electrochemical gradient.		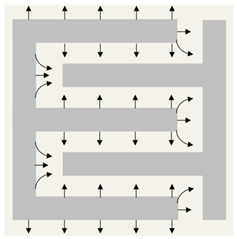 Silver rectangles represent silver electrodes, beige represents the inert dressing, and black arrows represent the electrochemical gradient.	

## Data Availability

Not applicable.
